# Sleeping Beauty transposon integrates into non-TA dinucleotides

**DOI:** 10.1186/s13100-018-0113-8

**Published:** 2018-02-07

**Authors:** Yabin Guo, Yin Zhang, Kaishun Hu

**Affiliations:** Guangdong Provincial Key Laboratory of Malignant Tumor Epigenetics and Gene Regulation, Medical Research Center, Sun Yat-sen Memorial Hospital, Sun Yat-sen University, Guangzhou, 510120 China

**Keywords:** Sleeping beauty, Transposon, Integration, Non-TA dinucleotide, Asymmetric, Non-palindromic

## Abstract

**Background:**

Sleeping Beauty transposon (SB) has become an increasingly important genetic tool for generating mutations in vertebrate cells. It is widely thought that SB exclusively integrates into TA dinucleotides. However, this strict TA-preference has not been rigorously tested in large numbers of insertion sites that now can be detected with next generation sequencing. Li et al. found 71 SB insertions in non-TA dinucleotides in 2013, suggesting that TA dinucleotides are not the only sites of SB integration, yet further studies on this topic have not been carried out.

**Results:**

In this study, we re-analyzed 600 million pairs of Illumina sequence reads from a high-throughput SB mutagenesis screen and identified 28 thousand SB insertions in non-TA sites. We recovered some of these non-TA sites using PCR and confirmed that at least a subset of the insertions at non-TA sites are real integrations. The consensus sequence of these non-TA sites shows an asymmetric pattern distinct from the symmetric pattern of the canonical TA sites. Perfect similarity between the downstream flanking sequence and SB transposon ends indicates there may be interaction between the transposon DNA binding domain of transposase and the target DNA.

**Conclusion:**

The TA-preference of SB transposon is not as strict as what people had thought. And the SB integrations at non-TA sites might be guided by the interaction between the transposon DNA binding domain of SB transposase and the target DNA.

**Electronic supplementary material:**

The online version of this article (10.1186/s13100-018-0113-8) contains supplementary material, which is available to authorized users.

## Background

The Sleeping Beauty transposon (SB) is a DNA transposon of the Tc1/mariner family, which was constructed from a consensus transposable element sequence in the genome of the Salmonid subfamily of fish [1]. SB is capable of transposing in mammalian systems and has become a popular genetic tool for generating genome-wide mutations [2–5]. DNA transposons often have strong preferences for their target sites. For example, piggyBac strictly integrates into TTAA sites [6], while Hermes prefers T at the second position and A at the seventh position of its target site duplication (TSD) [7–9]. It has long been accepted that SB, along with all other transposons of the Tc1/mariner family, integrate only into TA dinucleotides, based on previous studies with limited integration events [10]. In 2005, Yant et al. identified more than 1300 SB insertions, which all targeted to TA dinucleotides [11], further confirming the strict TA preference of SB integration. With the advent of next generation sequencing, even more SB integration sites have been sequenced in the context of transposon-mediated mutagenesis assays. However, rather than examining for other preferences in SB integration, most studies have rejected non-TA integration sites as probable artifacts, discarding reads lacking TAat the start [3, 4]. In 2013, Li et al. identified 71 SB insertions at non-TA sites (~ 1.6% of the total insertions), raising the idea that TA dinucleotides may not be the sole targets of SB [12]. Recently, the largest-to date ex vivo SB mutagenesis screen was performed [5], in which more than 1100 integration libraries were sequenced, 600 million pairs of sequence reads were obtained, and 2 million SB target positions were identified, providing a uniquely rich dataset to mine for rare integration events. In this study, we re-analyzed these sequence reads and found that SB does in fact integrate into non-TA sites at a frequency of ~ 1.4%. Further analysis suggests that the SB insertions at non-TA sites might be a result of side reaction of the canonical integration.

## Results

### Twenty-eight thousand integrations at non-TA sites were identified by re-analyzing previous sequencing data

**Fig. 1:**
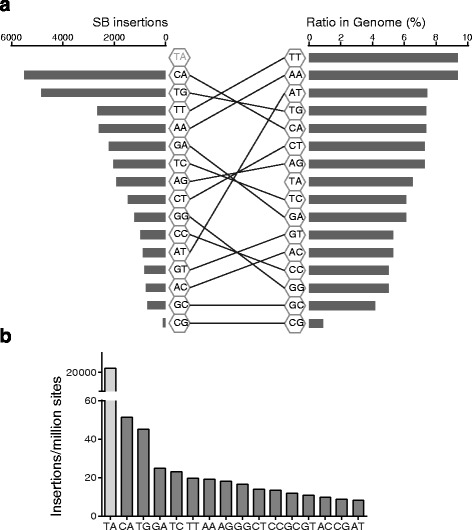
Count of SB insertions at non-TA dinucleotides and the frequency of dinucleotides in the mouse genome. **a** The order of the dinucleotides ranked by SB insertions (*general matches*) is distinct from the order of their frequency in the mouse genome. **b** Count of dinucleotides ranked by SB insertion rate, normalizing by the frequency of dinucleotides occurring in the mouse genome (only dinucleotides in non-repeat regions were included)

To search for possible integrations in all genomic contexts besides TA sites, we re-trimmed the sequence reads from a previous study that sequenced 600 million read pairs from SB integration libraries [5], including reads that began with non-TA sequences as well as TA dinucleotides. After aligning to the mouse genome (mm10), 2,018,489 unique sites were identified, of which 28,794 insertions were at non-TA dinucleotides (Additional file 1: Table S1 and Figure S1). Sequence reads from the same libraries that were aligned to the same coordinate and same strand were considered duplicates as previously described [13]. Briefly, duplicates comprise sequence reads from independent insertions, cell duplications, as well as PCR amplifications. The insertions at non-TA sites were roughly 1.4% of the total insertions, which is similar to the frequency reported previously [12]. These insertions were termed *general matches* (Table 1 and Fig. 1a). To be more stringent, we assumed that those non-TA dinucleotides could be sequencing errors of TA dinucleotides. We replaced the first two nucleotides of the sequence reads with TA, and aligned them to the mouse genome again. All the sequences that aligned successfully were removed from the *general matches* with the remaining inserts resulting in a new set termed *high stringency matches*. The *high stringency matches* are 92.8% of *general matches* (Table 1), suggesting that sequencing error cannot account for most detected insertions in non-TA contexts.

**Table 1:** Number of SB insertions identified at all 15 non-TA dinucleotides

Dinucleotides	General match	High stigency match
CA	5511	4916
TG	4853	3963
TT	2659	2366
AA	2600	2536
GA	2214	2168
TC	2046	1929
AG	1913	1907
CT	1478	1464
GG	1213	1211
CC	977	970
AT	894	890
GT	836	832
AC	767	750
GC	719	717
CG	114	112
Total	28,794	26,731

We found that the integration frequencies at different dinucleotides are distinct, ranging from 0.0055% to 0.28% (Table 1). To identify dinucleotides enriched in SB insertions, we compared the distribution of insertions with the distribution of dinucleotides in the mouse genome (Fig. 1a, Additional file 1: Table S2). For some dinucleotides, the frequency of insertions mirrored the dinucleotide frequency. For example, CG dinucleotides are very rare in mammalian genomes, and CG dinucleotides had the fewest insertions. However, the order of dinucleotides ranked by SB insertions is distinct from that ranked by their occurrences in the mouse genome (Fig. 1a), indicating that some dinucleotides are more preferred by SB integration than others. Normalizing SB insertions by the frequency of the dinucleotides in the mouse genome, we observed TG/CA dinucleotides as the most preferred non-TA sites of SB integration (Fig. 1b), which may be because they are the most similar dinucleotides to TA (only one transition from TA).

### A subset of integrations at non-TA site were validated using PCR

**Fig. 2:**
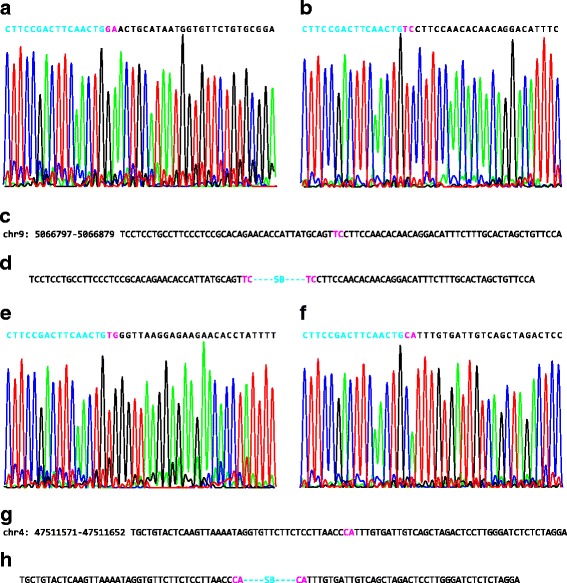
Recovery of SB integrations at non-TA sites. The SB integration site sequences were amplified in both orientations, using PCR and Sanger sequenced for integrations from lib155.11 (**a**, **b**) and lib133.13 (**e**, **f**). **c** and **g** The genomic sequences of the target sites. **d** and **h** The sequence patterns after integration. The cyan characters are the SB ends ((**a**) and (**e**) are right ends; (**b**) and (**f**) are left ends). The black characters are genomic sequences and; the pink characters are the TSDs

To confirm whether the SB insertions at non-TA sites are real integrations or artifacts introduced by experimental design or data analysis, we picked nine insertion sites for PCR amplification. Since each library is a pool of cell clones with different integrations, only insertions highly represented in the population (insertions with high duplicates, Additional file 1: Table S1) were possible to be recovered (Additional file 1: Tables S1 and S3). For each insertion, two PCR reactions were performed, one for each strand orientation. Four out of nine insertions showed clear single bands in agarose gels for both primer pairs, which were then sequenced by Sanger sequencing. Figure 2 shows two sites recovered from lib155.11 and lib133.13. Junctions between transposon and genomic sequences were found in both directions and perfect TSD was identified for each site (Fig. 2). This result indicates at least some of the non-TA sites found in sequence analysis are bona fide integrations.

However, TSDs were not identified at other sites (Additional file 1: Figure S2). For example, the TA dinucleotides are preserved to the right, but not to the left of the SB insertion at a site in lib165.5 and lib160.12, thus no TSD was formed. These sites may be the result of aberrant integrations, which has described in HIV-1 previously [14]. Because the LM-PCR for Illumina sequencing only detects the SB left end [5], our analysis cannot distinguish non-TA sites integrations from these aberrant integrations. To see if there are many sequences with patterns resembling what shown in Additional file 1: Figure S2, We examined the genomic sequences at all the non-TA sites and found that 1922 sequences have TA immediately after the target dinucleotides, while 1204 sequences have TA at the second position after the target dinucleotides. Even if the combined 3126 sites were the result of aberrant integrations, they are still only 0.1% of the total insertions at non-TA sites. Therefore, aberrant integrations do not contribute significantly to the insertions at non-TA sites identified in our analysis.

### The target site sequences of non-TA sites have an asymmetric pattern

**Fig. 3:**
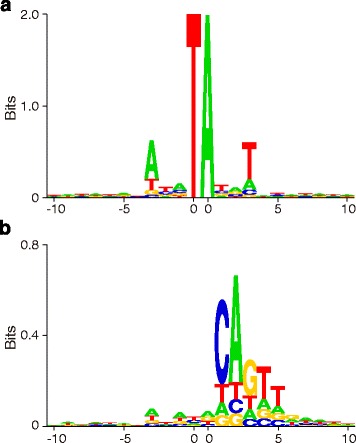
Sequence preferences at SB integration sites. The target site sequences were aligned according to the SB target site and orientation. **a** Sequences of all target sites (*n* = 2,018,489); (**b**), sequences of non-TA target sites (*n* = 28,794)

To find the sequence pattern of SB target sites, we extracted the SB target site sequences from the mouse genome by their coordinate and strand, and displayed the sequence preferences as sequence logos (Fig. 3). Like many transposons/retrotransposons, the SB target site sequences show a perfect symmetric pattern (palindrome) (Fig. 3a). Recently, Kirk et al. stated that the palindromic consensus sequence at the target sites of some retroviruses is a result of integrations occurring “in approximately equal proportions on the plus strand and the minus strand of the host genome” [15]. However, the symmetric sequence logos in some previous studies on transposon/retrotransposon [8, 16], as well as the present study, were all made of sequences with fixed orientation (i.e. reverse complement sequences were taken for integrations in the minus strand). Actually, in Sleeping Beauty and Hermes transposon, or Tf1 retrotransposon, the consensus sequences at target sites are always palindromic, even if the sequence logos are made of sequences from plus or minus strand separately (data not shown).

**Fig. 4:**
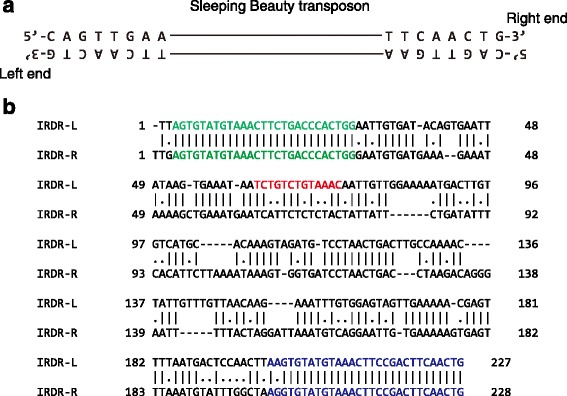
**a** The sequences of left end and right end of the Sleeping Beauty transposon. **b** The alignment of the SB IRDR-L and IRDR-R. The green characters are inner DRs; the blue characters are out DRs; and the red characters are HDR in left IR only

We then generated sequence logos with target site sequences only from non-TA sites of *general matches*(Table 1). Strikingly, a distinct asymmetric pattern was revealed (Fig. 3b). The upstream sequence flanking the insertion is essentially unchanged from that of the canonical TA sites (Fig. 3a), whereas the downstream sequence shows a conserved motif, CAGTTGAA. Interestingly, this consensus sequence is exactly the same as the sequence of the SB transposon ends (Fig. 4). We also made sequence logos with sequences from different dinucleotides separately, and they all showed a similar pattern (Additional file 1: Figures S3-S5). To our knowledge, this is the first time that a target site consensus sequence has been shown to replicate a transposon sequence.

## Discussion

We analyzed 600 million pairs of Illumina sequence reads, allowing for reads with all other dinucleotides as well as TA dinucleotides, and identified 28 thousand SB insertions at non-TA dinucleotides, which is around 1.4% of the total insertions. These insertions were not randomly distributed at non-TA dinucleotides. On the contrary, they showed preferences for different dinucleotides,CA/TG being the most preferred ones (Fig. 1). To confirm that these integrations are real, we recovered some integration sites using PCR from the genomic DNA of certain integration libraries. Sanger sequencing showed that two out of four sites have perfect TSDs, indicating they are bona fide integrations (Fig. 2). We also identified two aberrant integrations (Additional file 1: Figure S2). Accounting for aberrant integrations and possible sequencing errors, we infer that the frequency of SB integration into non-TA sites is at least 1%.

We confirmed that Sleeping Beauty transposon can integrate into non-TA dinucleotides using large number of insertions and a PCR recovery assay, thus modifying the well-established strict TA-preference of SB integration. Although ignoring these integration events, which account for ~ 1% of total integrations, does not change the conclusions of previous studies of SB integration, these low-frequency events have important consequences nonetheless. Recent studies have explored the use of SB in gene therapy [17, 18]. Rare integrations in non-TA sites need to be considered in these experiments to minimize the possibility of unexpected insertions disrupting gene functions.

Unlike the general SB target sites, which have a palindromic consensus sequence, these non-TA sites show a distinct non-palindromic pattern. The consensus sequence downstream of the target site sequence (5’-CAGTTGAA-3′) is exactly the same sequence as the SB transposon end. We considered if the identical sequence pattern of the consensus sequence at these target sites and the transposon end is an artifact: 1) the LM-PCR detects the junction between SB left end and genomic DNA, but the consensus sequence pattern is to the right side of the target site; 2) the target site sequences are extracted from the mouse genome, but not from sequence reads of Illumina sequencing; 3) there are no homologous sequences of SB in the mouse genome; thus, even if the SB sequences were amplified in LM-PCR, they still could not be aligned to the mouse genome (although there are CAGTTGAA sequences in the mouse genome, they are not long enough for alignment); 4) the consensus sequence is a sequence of most frequent nucleotides, but is not necessarily a real sequence. Therefore, it is highly unlikely the detected sequence pattern is an artifact.

Moreover, the insertions at non-TA sites are not due to homologous recombination, because: 1) the consensus sequence is not long enough for homologous recombination; 2) the insertions have strong orientation bias (strand bias) (Fig. 3b); and 3) the TSDs found in PCR (Fig. 2) indicate true integration events.

We then focus on the 8-nucloetide box adjacent to the target dinucleotide at the right side of the target sites (called the R8 box), where the consensus sequence is located. Among the 28,794 non-TA sites, 12,732 (44%) R8 boxes are CAGnnnnn, 6276 (22%) R8 boxes are CAGTTnnn, and 694 (2.4%) R8 boxes are CAGTTGAA. When the insertion numbers were normalized by the occurrence of the sequences in the mouse genome, we observed that the integration efficiency increased dramatically as the similarity between the R8 box sequence and the transposon end increased (Additional file 1: Figure S6). The frequency of SB integration in the context of the CAGTTGAA motif is > 12,000/million sites, more than half of the integration frequency at canonical TA sites (Fig. 1b). It is possible that the low number of integrations at non-TA sites is not due to low integration efficiency at non-TA sites, but because there are far fewer CAGTTGAA sites than TA dinucleotides in the mouse genome.

Since the occurrence frequency for an 8-nucleotide sequence is roughly 4^− 8^, the parity between these two sequences could not be a coincidence. Instead, it is far more likely to be a result of specific interaction. Therefore, we hypothesize that when certain genomic DNA sequences are similar to the SB end sequences, the transposon DNA binding domain of one SB transposase molecule in the pre-integration complex (PIC) may bind these genomic DNA strands as if they are transposon DNA strands, thus guiding the PIC to integrate into these positions, even there are no TA dinucleotides. This process might be a side reaction resembling the aberrant integration described recently by Wang et al. [19], in which the transposon DNA is circularized with one end attached to the other. Due to the small contribution of the side reaction to the entire integration collection, only when it is viewed separately from the canonical integration, can we notice its different property.

Why is there consensus sequence only at the downstream of the target dinucleotides, but not at both sides? SB is a transposon of the inverted repeat direct repeat (IRDR) subfamily [10, 20]. IRs are located at the two ends of the transposon. Each IR contains two DRs for binding transposase. However, the two IRs are asymmetric. A half direct repeat motif (HDR) which also can binds transposase was only found in the left IR (Fig. 4b). This could be the reason that only the transposon DNA binding domain of the transposase at the one side is capable of interacting with genomic DNA strands.

Notably, CA/TG are the most similar dinucleotides to TA, while the sequence pattern flanking them are the weakest (Additional file 1: Figures S3-S5), which indicates that there is no absolute barrier between the side reaction and the canonical reaction. The insertions at non-TA sites should be a pool of both canonical reaction and side reaction. When the target dinucleotides are more different from TA, the integration will rely more on the interaction between the DNA binding domain of transposase and the target DNA.

Finally, the SB transposase used for generating the present integrations is the hyperactive SB100X [21], and the SB transposase used by Li et al. is another hyperactive version, HSB16 [12, 22]. Probably, hyperactive versions of transposase tend to have less strict preference, which is to be answered by future studies.

## Conclusion

We have shown that SB transposon integrates into non-TA sites in addition to TA sites and suggest that these integrations are guided by interactions between SB transposase and genomic DNA sequences resembling the sequence of transposon end. Our finding improved the knowledge on the strict TA-preference of SB transposon.

## Methods

### Data source

The generation of the SB integration libraries in mouse BaF3 cells with SB100X transposase [21] and T2/Onc vector were described previously [5]. The Illumina sequencing results were deposited in NCBI Short Read Archive, http://www.ncbi.nlm.nih.gov/sra. Accession no. SRX1491647.

### Bioinformatic analyses

Scripts for sequence trimming and dinucleotide frequency counting were written in Perl. The trimmed sequences were aligned to the mouse genome (mm10) using Bowtie 2 [23]. The alignment output was filtered using a Perl script.

The target site sequences were extracted from the mouse genome using a Perl script. Reverse complement sequences were taken when the integration orientations are right-to-left (i.e. at minus strand). The target site sequence logos were generated using an application called DNAlogo [24], which has been described in previous studies [8, 16]. The output PostScript (.ps) vector maps were converted to .pdf format in Adobe Illustrator.

### Recovering the SB target sites by PCR

Genomic DNA samples which had been extracted from the cell pools of SB integration libraries using DNeasy Blood & Tissue Kit (Qiagen) were obtained from Dr. Kathryn O’Donnell’s lab at UT Southwestern Medical Center. In this study, the genomic DNA samples were used as templates. Primers were designed according to the genomic sequences flanking the SB target sites and the SB left/right ends. The primer pairs are [Primer 5, SB-left] and [SB-right, Primer 3] for insertions at plus strand, or [Primer 5, SB-right] and [SB-left, Primer 3] for insertions at minus strand (Additional file 1: Table S3). PCR reactions were performed using CloneAmp HiFi PCR Premix (Clontech). The PCR products were then sequenced by Sanger sequencing.

## Additional file

Additional file 1: Supplementary tables and figures. (ZIP 3928 kb)

## Abbreviations

IRDR: Inverted repeat direct repeat
PIC: Pre-integration complex
TSD: Target site repeat
