# Supplementary material for: Sleeping Beauty transposon integrates into non-TA dinucleotides
Source: Mob DNA. 2018 Feb 7;9:8. doi: 10.1186/s13100-018-0113-8 (PMC5801840; doi:10.1186/s13100-018-0113-8)
Supplement: Additional file 1: — Supplementary tables and figures. (ZIP 3928 kb) [file 13100_2018_113_MOESM1_ESM.zip › Supplementary Materials.pdf]

SUPPLEMENTARY MATERIALS

Sleeping Beauty transposon integrates into non-TA dinucleotides via an alternative mechanism

Yabin Guo, Yin Zhang and Kaishun Hu

From the Guangdong Provincial Key Laboratory of Malignant Tumor Epigenetics and Gene Regulation, Medical Research Center, Sun Yat-sen Memorial Hospital, Sun Yat-sen University, Guangzhou, 510120 China.

CONTENTS

Supplementary table S1-S3

Supplementary figure S1-S6

Supplementary tables

Table S1.

28,794 unique insertions at non-TA sites were identified. The chromosome, coordinate, strand, library, target site dinucleotide and total sequence reads were shown (see separated Excel file).

Table S2.

The ratios of all 16 dinucleotides occurring in the mouse genome (repeat regions were excluded, since the SB insertions in repeat regions could not be identified).

Dinucleotides	Frequency (%)
TT	9.368
AA	9.362
AT	7.456
TG	7.393
CA	7.392
CT	7.286
AG	7.282
TA	6.532
TC	6.116
GA	6.111
GT	5.297
AC	5.294
CC	5.019
GG	5.016
GC	4.169
CG	0.905

Table S3.

Target sites recovery using PCR. Non-TA sites with high duplicates were chosen. Primers were designed according to the genomic sequences flanking the target sites (see separated Excel file).

Figure S1. The distribution of 28,794 SB insertions on the mouse chromosomes. The insertions numbers per 100 kb interval were shown.

Figure S2. The identification of aberrant SB integrations. The SB integration site sequences were amplified in both orientations, using PCR and Sanger sequenced for integrations from lib155.11 (A and B) and lib133.13 (E and F). C and G, the genomic sequences of the target sites. D and H, the sequence patterns after integration. The cyan characters are the transposon sequences (A and E are right ends; B and F are left ends); the black characters are genomic sequences; the pink characters are the dinucleotides adjacent to the transposon ends and; the gray characters in parentheses are the nucleotides that should have occurred if the integrations are concerted instead of aberrant (D and H)

Figure S3. The sequence patterns at different non-TA target sites. A-F: CA, TG, GA, TC, AA, TT.

Figure S4. The sequence patterns at different non-TA target sites (continued). A-F: AG, CT, AC, GT, GG, CC.

Figure S5. The sequence patterns at different non-TA target sites (continued). A-C: GC, CG, AT.

Figure S6. Number of SB insertions and R8 boxes. The number of SB insertions increased dramatically as the similarity between R8 box and the transposon end increased

Figure S1

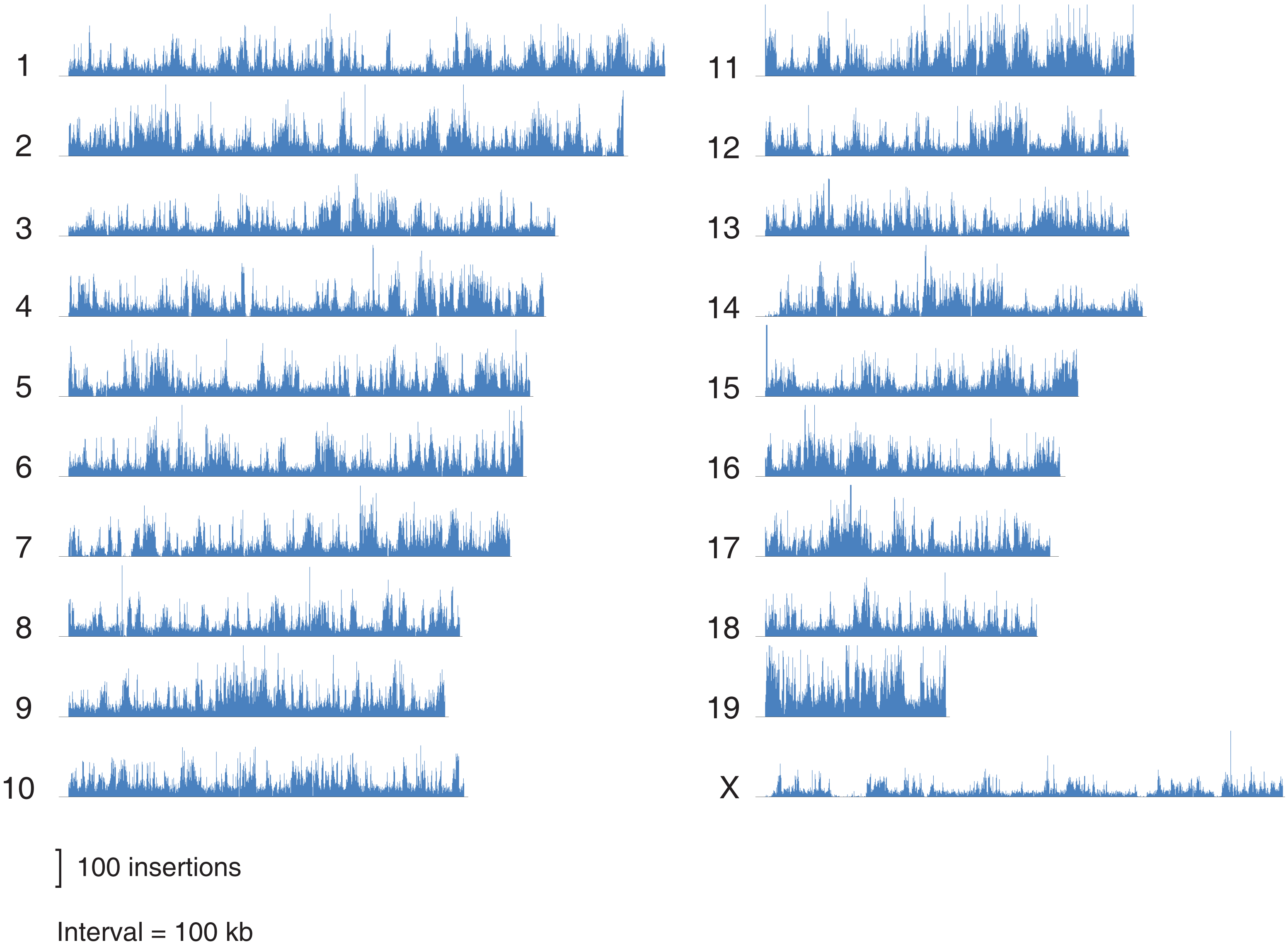
1
2
3
4
5
6
7
8
9
10
11
12
13
14
15
16
17
18
19
X
100 insertions
Interval = 100 kb

Figure S2

A

CTTCCGACTTCAACTGTATCACTATTTTGTTGTTTACTTATTT

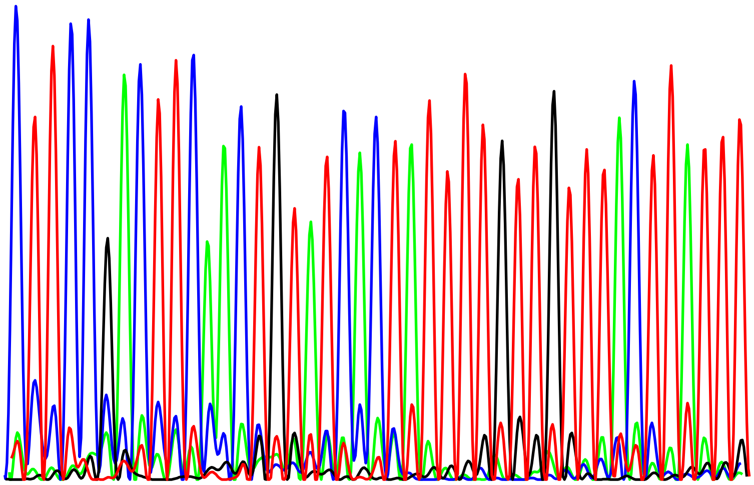

B

CTTCCGACTTCAACTGCTGTAGAGAGGCAGACATAATTATAAT

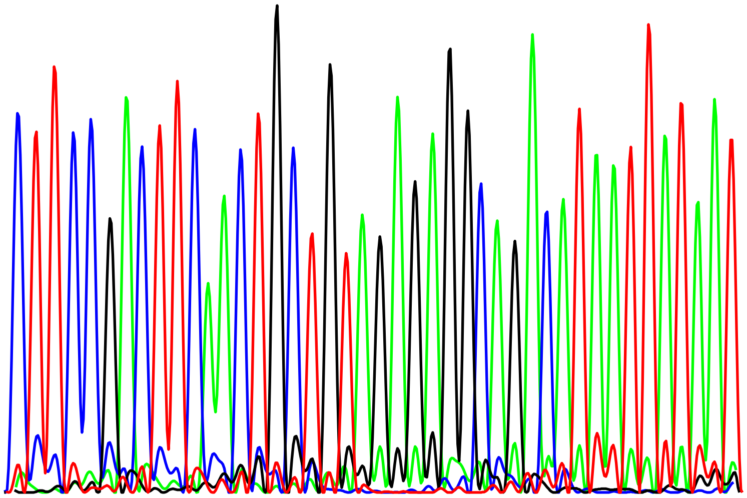

C

chr11: 39854505-39854589 AGTGATCAATAATGCAAATAAGTAAACAACAAAATAGTGATAACTGTAGAGAGGCAGACATAATTATAATGTCTTCTAGAAGCAT

D

AGTGATCAATAATGCAAATAAGTAAACAACAAAATAGTGATA----SB----(TAA)CTGTAGAGAGGCAGACATAATTATAATGTCTTCTAGAAGCAT

E

CTTCCGACTTCAACTGTACATGAACATTTCCCCCTTGTAAT

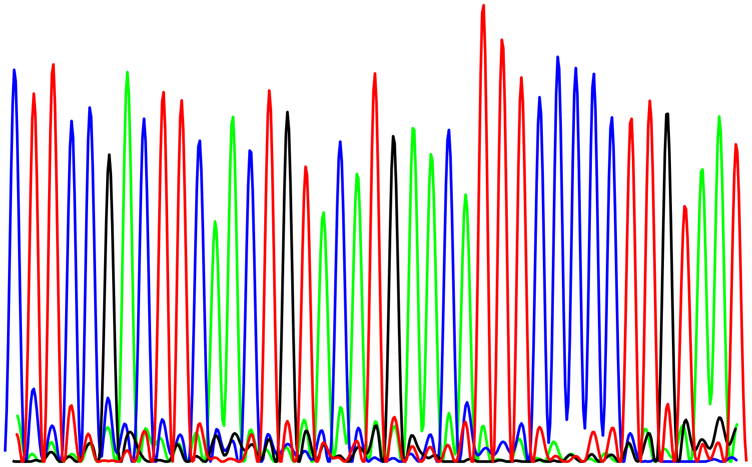

F

CTTCCGACTTCAACTGCAGTGACTATGAAATAATCAATATTTC

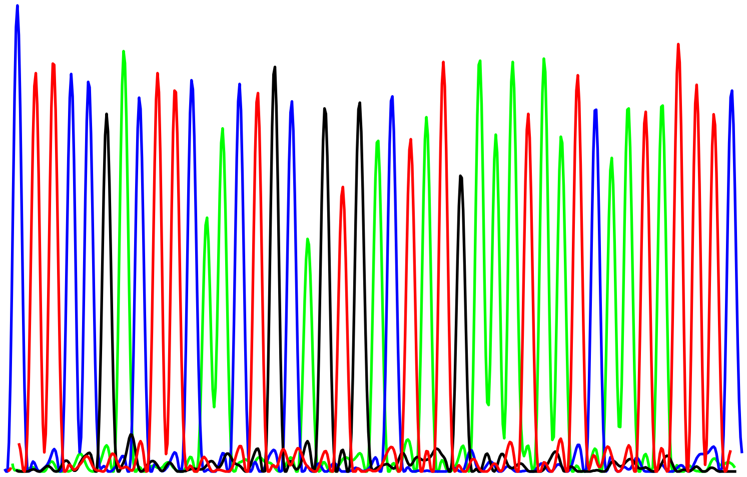

G

chrX: 50631131-50631214 CAGCTACCAACACCACCATTACAAGGGGGAAATGTTCATGTACAGTGACTATGAAATAATCAATATTTCTTACTATGCTGACAC

H

CAGCTACCAACACCACCATTACAAGGGGGAAATGTTCATGTA----SB----(TA)CAGTGACTATGAAATAATCAATATTTCTTACTATGCTGACAC

Figrue S3

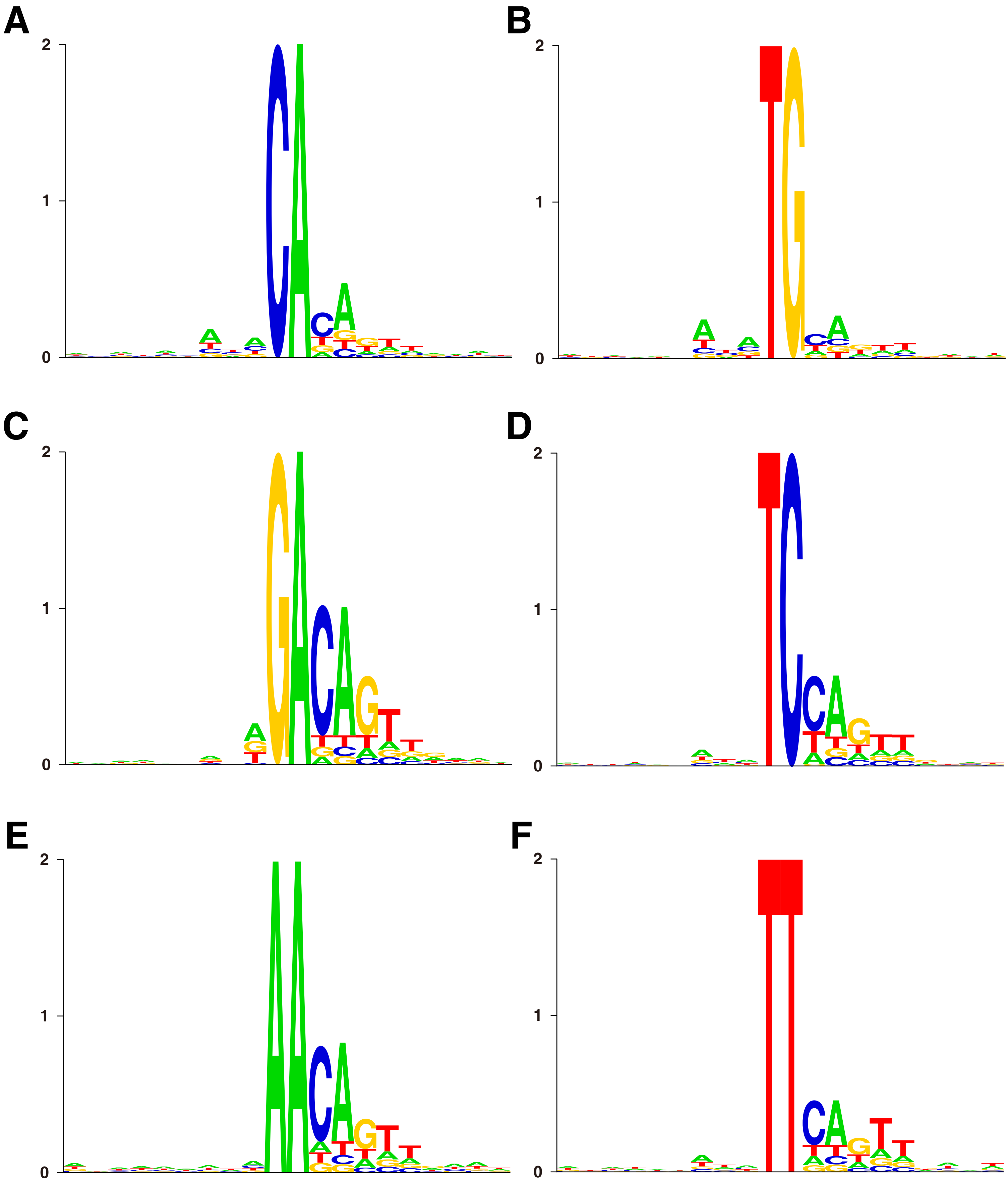

Figure S4

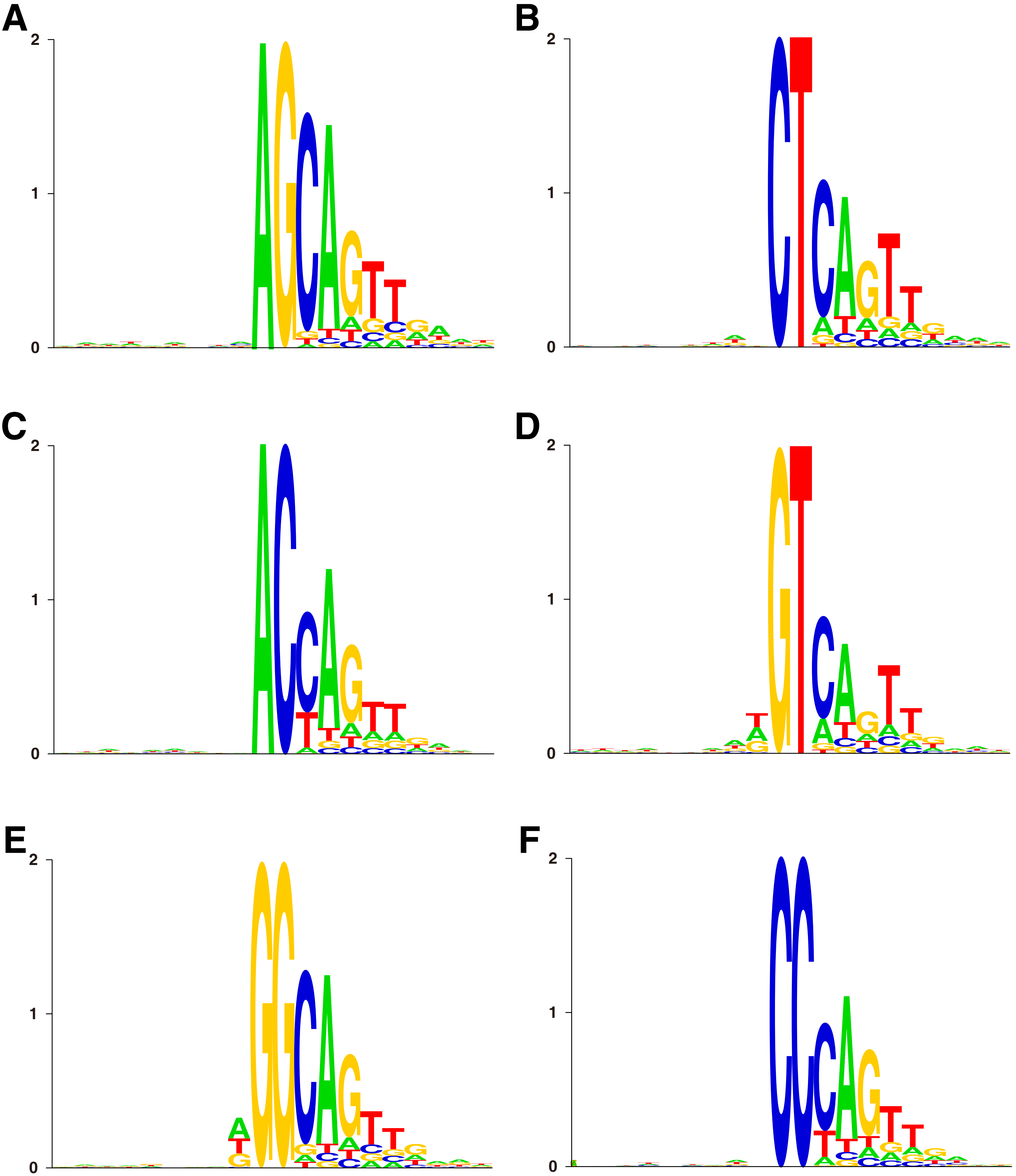

Figure S5

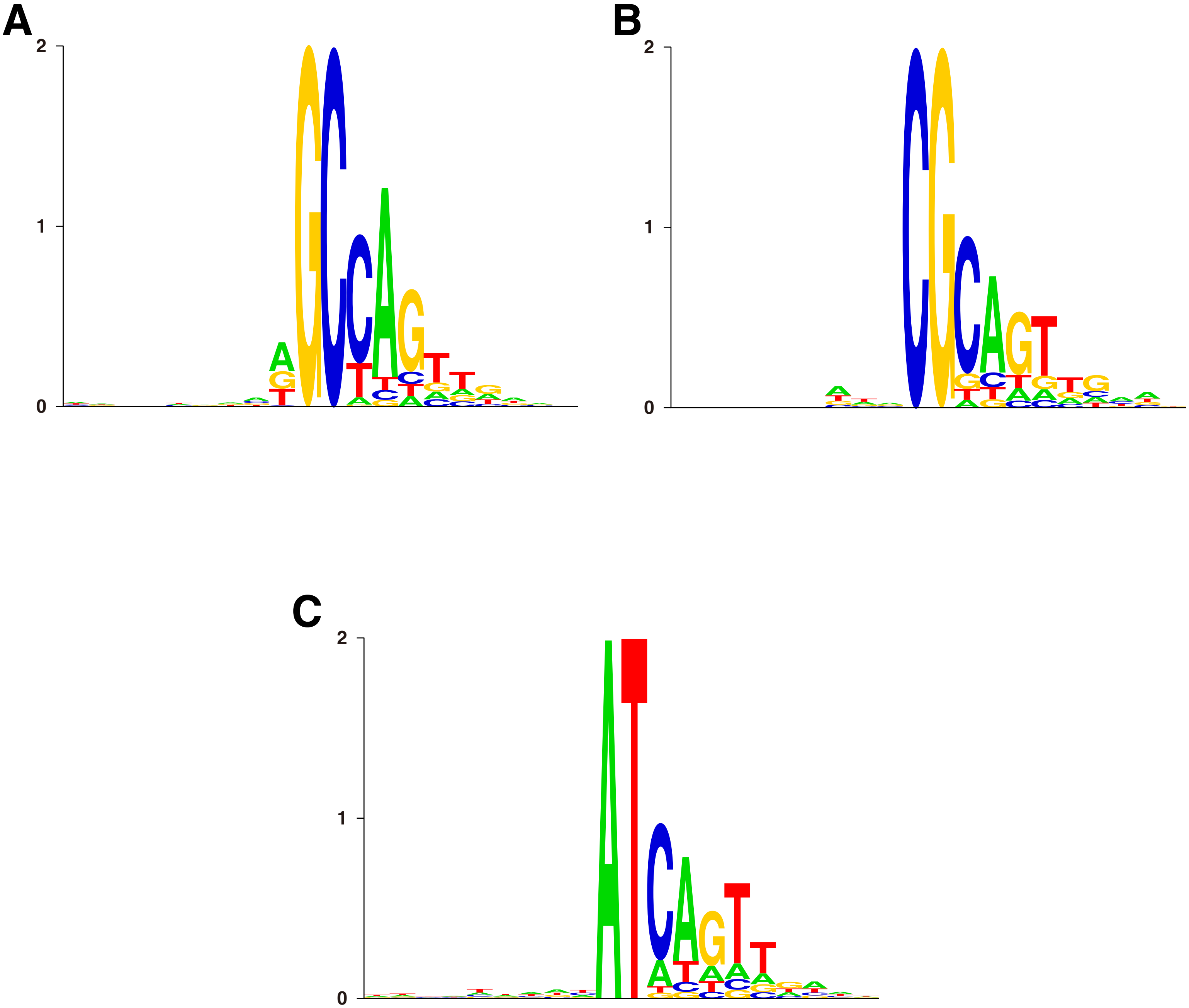

Figure S6

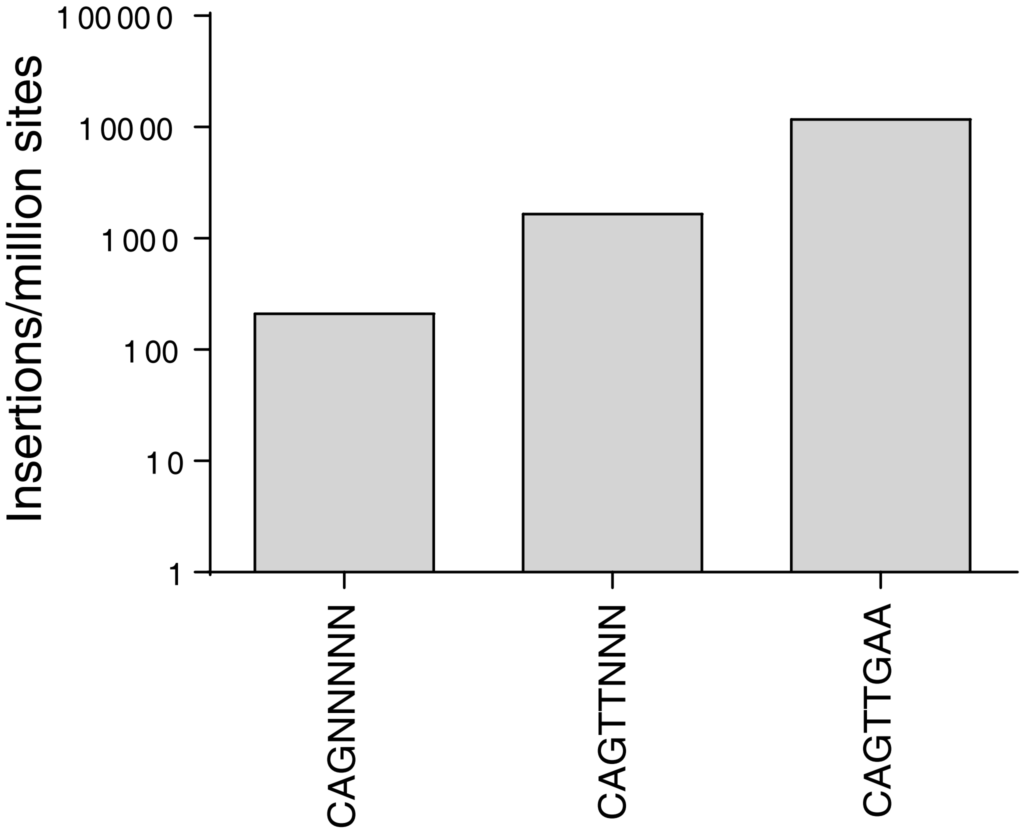

Insertions/million sites
100000
10000
1000
100
10
1
CAGNNNNN
CAGTTNNN
CAGTTGAA
